# Supplementary material for: High‐Throughput In Vivo Screening Using Barcoded mRNA Identifies Lipid Nanoparticles With Extrahepatic Tropism for In Situ Immunoengineering
Source: Adv Mater. 2026 Jan 28;38(13):e14370. doi: 10.1002/adma.202514370 (PMC12957869; doi:10.1002/adma.202514370)
Supplement: Supplementary file 1 — Supporting File: adma72049‐sup‐0001‐SuppMat.pdf. [file ADMA-38-e14370-s001.pdf]

Supporting information: High-throughput in vivo screening using barcoded mRNA identifies lipid nanoparticles with extrahepatic tropism for in situ immunoengineering

*Alex G. Hamilton Ajay S. Thatte Junchao Xu Zhangyi Luo Hannah C. Safford Kelsey L. Swingle Jenna Muscat-Rivera Michael Kegel Xuexiang Han Ryann A. Joseph Amanda M. Murray Hannah C. Geisler Ricardo C. Whitaker Lulu Xue Roman Spektor Jilian R. Melamed Drew Weissman Michael J. Mitchell**

Dr. A.G. Hamilton, A.S. Thatte, Dr. J. Xu, Dr. Z. Luo, H.C. Safford, Dr. K.L. Swingle, Dr. X. Han, R.A. Joseph, A.M. Murray, H.C. Geisler, Dr. R.C. Whitaker, Dr. L. Xue
Department of Bioengineering, University of Pennsylvania, Philadelphia, PA 19104, USA

J. Muscat-Rivera, M. Kegel, Prof. J.R. Melamed
Department of Medicine, Perelman School of Medicine, University of Pennsylvania, Philadelphia, PA 19104, USA

Dr. R. Spektor
Field of Genetics, Genomics, and Development, Cornell University, Ithaca, NY 14853, USA

Prof. D. Weissman
Department of Medicine, Perelman School of Medicine, University of Pennsylvania, Philadelphia, PA 19104, USA
Penn Institute for RNA Innovation, University of Pennsylvania, Philadelphia, PA 19104, USA

Prof. M.J. Mitchell*
Department of Bioengineering, University of Pennsylvania, Philadelphia, PA 19104, USA
Penn Institute for RNA Innovation, University of Pennsylvania, Philadelphia, PA 19104, USA
Abramson Cancer Center, Perelman School of Medicine, University of Pennsylvania, Philadelphia, PA 19104, USA
Institute for Immunology, Perelman School of Medicine, University of Pennsylvania, Philadelphia, PA 19104, USA
Cardiovascular Institute, Perelman School of Medicine, University of Pennsylvania, Philadelphia, PA 19104, USA
Institute for Regenerative Medicine, Perelman School of Medicine, University of Pennsylvania, Philadelphia, PA 19104, USA
Email Address: mjmitch@seas.upenn.edu

List of Supplementary Tables

1 Top 200 barcode sequences used to produce an initial library of b-mRNAs for in vivo evaluation. 7
2 Final 134 barcode sequences established as suitable for in vivo mRNA LNP screening. . . 10
3 Formulation details of tested LNP library. 13
4 Antibody clones used for flow cytometry and FACS. 14

List of Supplementary Figures

1 Hydrodynamic diameter of C12-494 LNPs containing b-mRNA pool. 15
2 Plug-and-play reaction principle. 16
3 Representative flash purification traces (ELS detection). 17
4 Mass spectrum of 17D9.2 (ESI, loop injection). 18
5 Mass spectrum of 18D4 (ESI, loop injection). 19
6 Mass spectrum of 20D8i (ESI, loop injection). 20
7 ^{1}H NMR (400 MHz) spectrum of 17D9.2 in $CDCl_3$. 21
8 ^{1}H NMR (400 MHz) spectrum of 18D4 in $CDCl_3$. 22
9 ^{1}H NMR (400 MHz) spectrum of 20D8i in $CDCl_3$. 23
10 Physicochemical characterization of b-mRNA LNP library. 24
11 Representative blood/spleen flow cytometry gating scheme. 25
12 Representative lung flow cytometry gating scheme. 26
13 Representative liver flow cytometry gating scheme 26
14 Ionizable lipid structure-function relationship. 27
15 Body weight change of mice inoculated with B16-OVA melanoma cells. 28
16 Tumor growth curves from mice inoculated with B16-OVA melanoma cells. 28
17 Protein adsorption to SORT LNPs. 29

Barcode	Sequence
1	CCTCTTGTGTGG
2	AATGTTCTCTCC
3	GGAGAAGAAGAA
4	TATGGTTGTGTG
5	GGTCGGTGTTGT
6	CCTTCTGTTCTG
7	CTCTTCGCCTCT
8	TTCGGTTCTTCC
9	TTATCCTCTCCA
10	GTGTTCTCCTTA
11	TTCTCGGTGTGG
12	TTGTTGGCTGCC
13	CTTGGTGGTTCC
14	GGTCCTTCCTCA
15	TGGTGTGGTCTC
16	GTGGTTGGTTAA
17	GTGTTGTGTCCG
18	AACTCCTTCCGG
19	AATCCATCTTCC
20	TTCACACCACAA
21	AAGGAAGGTGGA
22	AAGTGGTTGCGG
23	CCAACACCAGCA
24	CCAAGGTGTGTG
25	GAGATTGTGTTG
26	GCGCCTTCTCTA
27	TCCTCCGCTTGT
28	CTTCCTTGTCGG
29	AATTATTGCCGG
30	CCAATTCTCCTA
31	TTATGATGGTGG
32	TGTGTCTTCGCC
33	GGAGTCTCTCTC
34	ACCTTCCTCTAA
35	AACCTCTCTCAA
36	TAGAGAGGAGAA
37	CCACACATTCAA
38	TAATCCTCCGCG
39	AGGCCACACCAA
40	GGCAATCCTCTT
41	ACAACCAATCCA
42	GACCACACATAA
43	TTGTGCGCGTTC
44	TCCGTTGTCTCC
45	CACCACTCATCC
46	AGAATAGGTTGG
47	TTCCTTCGTTGA
48	TGTTGTTGGAGA
49	CGGTTGTCCTGT
50	GGAGGAACACAA

Barcode	Sequence
51	TCCTCTTCACCG
52	ATAGTGGCGTGG
53	AAGAGGAGCGAA
54	AGGAAGAGGCAA
55	ATTGTGTGATGG
56	AACCACCGGCAA
57	ACACACAACCGA
58	TTCCGCCACTCA
59	CTTGTGGTGTAA
60	ACGCTGTTGTGG
61	AAGAACAAGCGG
62	TGGCTCTTCTCA
63	CTCCAACCTTAA
64	TGGTGGCGCTTA
65	ACAAGAGAGGTA
66	TTGGTGTATTGC
67	GGCCACCTTATA
68	AGGTGTTCTTGA
69	ACCTCTCTTGGA
70	TTATAGGCTTGG
71	TCCGTCTTGTTA
72	GCCATCTCCTCA
73	GGTTGTTGCTAA
74	CCTCCGTTCCAA
75	AACCAAGCCTAA
76	TCGTTCTGTGTG
77	CCGCGTTATTAA
78	AAGCAACCTCCA
79	CACTTCATTCTC
80	GTACAACACCAA
81	TGCCTCCATCCA
82	TAGAGGATGTGG
83	TTCTCCAATACC
84	GATTGCTTCTTG
85	AACACCGCTTAA
86	TGTGGAAGAGAA
87	ACACCGCTCTGT
88	GCCGCTTATTCC
89	GTCTCTTGGTGC
90	CTCGCTTATCTT
91	CATACTTCCTTG
92	TTCTTCCTGCAA
93	ATTACCTCGCGG
94	GGAGAGAATTGG
95	CATCGTGGTTGG
96	GGTGAGAGGTAA
97	AATAGGTAGTGG
98	CACCTAACACAA
99	TGGAACAACCAA
100	ACCACGGCTGTT

Barcode	Sequence
101	TCCAATCTTCTG
102	TCCTTCTACCTG
103	CCTTCTAACACC
104	GGTCTTGATCTT
105	AAGGCTCCTCAC
106	TGGAGGTAGTTG
107	GTGGAGTCTTCC
108	AGGTCACCTCTT
109	ACGGTGTGTTAA
110	CCTCACCACTTG
111	GTAACACTCTTC
112	GCCAATGGTTGG
113	GTTGGCCTTGTA
114	TAACACAGCCAA
115	CGTTCCTCTTGA
116	GTCCTCTGTTCG
117	TCTCCACTCTAA
118	TACACTTGTTGG
119	CTTCTTACGCGG
120	ACCAGCCACCTA
121	GGTTGCTTATCC
122	AAGAACCACTTG
123	AGAGAGAGCCTA
124	TTGTGGTAGCGC
125	CCTCATTATTCG
126	TTACGCCTTCGC
127	ATACCTGTGTGG
128	ACCAACCTTGCA
129	CAAGGCCTTCCA
130	TCCATCCTATTC
131	ATTGGCGTCTGG
132	TCCTATTCGTGG
133	GAGTGGTGGTCA
134	CGGAAGAAGGTG
135	GGTTGGAGTGAA
136	CCGACTCTCTCA
137	CGCCACAACTCA
138	TGTGGCCTCCAA
139	GCCGAACAACAA
140	ACAGACTCTTCC
141	AACTGCTGGTGG
142	CATATATGGCGG
143	TCTGCTTCTCGG
144	GCTTACCTTCTA
145	CCGTTATATACC
146	CCTTGTTGTCAA
147	ACAACGCGCCAA
148	CAAGAACACTCA
149	GCTGTTCGTTCG
150	TCCACCTCCGAA

Barcode	Sequence
151	GTTGGAGGCGTA
152	GGTTATCTGCGG
153	TAGGAGCCTCCT
154	TCCAGGAGAGAA
155	ACACAGAGACAA
156	CCGGAGAATTAA
157	AACAGGAGGCTC
158	TGTTCAGTTCTC
159	GGTGGTCTATAA
160	GGTGTTCAGTGG
161	GGCATTGTTCTG
162	CAACCGTCGCTT
163	CAATAACCTTCG
164	TTCTTGGAGGAA
165	CGGTGGATGGAA
166	TATAGGAGACGG
167	CTTCCTACTCAA
168	ATGCCTCTTCGG
169	GAAGTGAGAGAA
170	AGAACGACACAA
171	AACGGCACACAA
172	CTTCTCCAGGTT
173	AGGACGGTTCTC
174	TATTCTGCCTCA
175	TCTGGTCTGCTT
176	GGAAGTTGTGAA
177	ACCTTGTATTCC
178	GAACACAAGGAA
179	TGGAACTGTGTT
180	CAAGAGAAGGCC
181	CTTGTTCATTGC
182	CACACCTCTGCA
183	TTCTCGTGCTTA
184	ACTTGCCTTCGG
185	CTTCCACTGTTA
186	AACAAGCGAGAA
87	ACGTGAGGTTGG
188	GGCACAACAGAA
189	AGAACACCTCGA
190	AATTAAGCTCCG
191	TAACACCGGTCG
192	AGGATGAGTGTG
193	GGAGTGGATATA
194	TAATGGAGGTGA
195	GGAAGGCATTAT
196	AGGTTATGGTGA
197	AGTATTCTCTGG
198	TATCCATTGTGG
199	CTTGTTCCACAA
200	CGAGCGGTGTTA

Barcode	Sequence

Supplementary Table 1: Top 200 barcode sequences used to produce an initial library of b-mRNAs for in vivo evaluation.

Barcode	Sequence	Index
4	TATGGTTGTGTG	1
5	GGTCGGTGTTGT	2
6	CCTTCTGTTCTG	3
7	CTCTTCGCCTCT	4
8	TTCGGTTCTTCC	5
9	TTATCCTCTCCA	6
10	GTGTTCTCCTTA	7
13	CTTGGTGGTTCC	8
14	GGTCCTTCCTCA	9
15	TGGTGTGGTCTC	10
17	GTGTTGTGTCCG	11
18	AACTCCTTCCGG	12
20	TTCACACCACAA	13
21	AAGGAAGGTGGA	14
23	CCAACACCAGCA	15
24	CCAAGGTGTGTG	16
25	GAGATTGTGTTG	17
26	GCGCCTTCTCTA	18
27	TCCTCCGCTTGT	19
29	AATTATTGCCGG	20
32	TGTGTCTTCGCC	21
39	AGGCCACACCAA	22
41	ACAACCAATCCA	23
44	TCCGTTGTCTCC	24
46	AGAATAGGTTGG	25
47	TTCCTTCGTTGA	26
49	CGGTTGTCCTGT	27
50	GGAGGAACACAA	28
51	TCCTCTTCACCG	29
53	AAGAGGAGCGAA	30
55	ATTGTGTGATGG	31
56	AACCACCGGCAA	32
57	ACACACAACCGA	33
58	TTCCGCCACTCA	34
59	CTTGTGGTGTAA	35
61	AAGAACAAGCGG	36
62	TGGCTCTTCTCA	37
64	TGGTGGCGCTTA	38
65	ACAAGAGAGGTA	39
66	TTGGTGTATTGC	40
68	AGGTGTTCTTGA	41
69	ACCTCTCTTGGA	42
71	TCCGTCTTGTTA	43
72	GCCATCTCCTCA	44
73	GGTTGTTGCTAA	45
74	CCTCCGTTCCAA	46
75	AACCAAGCCTAA	47
78	AAGCAACCTCCA	48
79	CACTTCATTCTC	49
80	GTACAACACCAA	50

Barcode	Sequence	Index
81	TGCCTCCATCCA	51
84	GATTGCTTCTTG	52
87	ACACCGCTCTGT	53
89	GTCTCTTGGTGC	54
90	CTCGCTTATCTT	55
91	CATACTTCCTTG	56
94	GGAGAGAATTGG	57
96	GGTGAGAGGTAA	58
97	AATAGGTAGTGG	59
98	CACCTAACACAA	60
99	TGGAACAACCAA	61
100	ACCACGGCTGTT	62
101	TCCAATCTTCTG	63
102	TCCTTCTACCTG	64
103	CCTTCTAACACC	65
104	GGTCTTGATCTT	66
105	AAGGCTCCTCAC	67
106	TGGAGGTAGTTG	68
107	GTGGAGTCTTCC	69
108	AGGTCACCTCTT	70
109	ACGGTGTGTTAA	71
110	CCTCACCACTTG	72
111	GTAACACTCTTC	73
112	GCCAATGGTTGG	74
113	GTTGGCCTTGTA	75
114	TAACACAGCCAA	76
115	CGTTCCTCTTGA	77
116	GTCCTCTGTTCG	78
118	TACACTTGTTGG	79
119	CTTCTTACGCGG	80
120	ACCAGCCACCTA	81
121	GGTTGCTTATCC	82
122	AAGAACCACTTG	83
123	AGAGAGAGCCTA	84
124	TTGTGGTAGCGC	85
125	CCTCATTATTCG	86
127	ATACCTGTGTGG	87
128	ACCAACCTTGCA	88
129	CAAGGCCTTCCA	89
130	TCCATCCTATTC	90
131	ATTGGCGTCTGG	91
133	GAGTGGTGGTCA	92
135	GGTTGGAGTGAA	93
136	CCGACTCTCTCA	94
138	TGTGGCCTCCAA	95
140	ACAGACTCTTCC	96
142	CATATATGGCGG	97
143	TCTGCTTCTCGG	98
146	CCTTGTTGTCAA	99
147	ACAACGCGCCAA	100

Barcode	Sequence	Index
149	GCTGTTCGTTCG	101
150	TCCACCTCCGAA	102
151	GTTGGAGGCGTA	103
153	TAGGAGCCTCCT	104
155	ACACAGAGACAA	105
157	AACAGGAGGCTC	106
158	TGTTCAGTTCTC	107
161	GGCATTGTTCTG	108
162	CAACCGTCGCTT	109
163	CAATAACCTTCG	110
165	CGGTGGATGGAA	111
166	TATAGGAGACGG	112
167	CTTCCTACTCAA	113
169	GAAGTGAGAGAA	114
171	AACGGCACACAA	115
172	CTTCTCCAGGTT	116
173	AGGACGGTTCTC	117
174	TATTCTGCCTCA	118
175	TCTGGTCTGCTT	119
176	GGAAGTTGTGAA	120
177	ACCTTGTATTCC	121
179	TGGAACTGTGTT	122
180	CAAGAGAAGGCC	123
181	CTTGTTCATTGC	124
182	CACACCTCTGCA	125
188	GGCACAACAGAA	126
189	AGAACACCTCGA	127
190	AATTAAGCTCCG	128
192	AGGATGAGTGTG	129
196	AGGTTATGGTGA	130
197	AGTATTCTCTGG	131
198	TATCCATTGTGG	132
199	CTTGTTCCACAA	133
200	CGAGCGGTGTTA	134

Supplementary Table 2: Final 134 barcode sequences established as suitable for in vivo mRNA LNP screening. Listed index numbers correspond to LNP formulation numbers.

Index	Ionizable lipid	%mol	Helper lipid	%mol	Cholesterol (%mol)	PEG-lipid	%mol
1	1D4	35	DOPE	16	46.5	C14-PEG2000	2.5
2	1D6.2	35	DOPE	16	46.5	C14-PEG2000	2.5
3	1D8	35	DOPE	16	46.5	C14-PEG2000	2.5
4	1D8i	35	DOPE	16	46.5	C14-PEG2000	2.5
5	1D18	35	DOPE	16	46.5	C14-PEG2000	2.5
6	1D9.2	35	DOPE	16	46.5	C14-PEG2000	2.5
7	2D4	35	DOPE	16	46.5	C14-PEG2000	2.5
8	2D6.2	35	DOPE	16	46.5	C14-PEG2000	2.5
9	2D8	35	DOPE	16	46.5	C14-PEG2000	2.5
10	2D8i	35	DOPE	16	46.5	C14-PEG2000	2.5
11	2D18	35	DOPE	16	46.5	C14-PEG2000	2.5
12	2D9.2	35	DOPE	16	46.5	C14-PEG2000	2.5
13	3D4	35	DOPE	16	46.5	C14-PEG2000	2.5
14	3D6.2	35	DOPE	16	46.5	C14-PEG2000	2.5
15	3D8	35	DOPE	16	46.5	C14-PEG2000	2.5
16	3D8i	35	DOPE	16	46.5	C14-PEG2000	2.5
17	3D18	35	DOPE	16	46.5	C14-PEG2000	2.5
18	3D9.2	35	DOPE	16	46.5	C14-PEG2000	2.5
19	4D4	35	DOPE	16	46.5	C14-PEG2000	2.5
20	4D6.2	35	DOPE	16	46.5	C14-PEG2000	2.5
21	4D8	35	DOPE	16	46.5	C14-PEG2000	2.5
22	4D8i	35	DOPE	16	46.5	C14-PEG2000	2.5
23	4D18	35	DOPE	16	46.5	C14-PEG2000	2.5
24	4D9.2	35	DOPE	16	46.5	C14-PEG2000	2.5
25	5D4	35	DOPE	16	46.5	C14-PEG2000	2.5
26	5D6.2	35	DOPE	16	46.5	C14-PEG2000	2.5
27	5D8	35	DOPE	16	46.5	C14-PEG2000	2.5
28	5D8i	35	DOPE	16	46.5	C14-PEG2000	2.5
29	5D18	35	DOPE	16	46.5	C14-PEG2000	2.5
30	5D9.2	35	DOPE	16	46.5	C14-PEG2000	2.5
31	6D4	35	DOPE	16	46.5	C14-PEG2000	2.5
32	6D6.2	35	DOPE	16	46.5	C14-PEG2000	2.5
33	6D8	35	DOPE	16	46.5	C14-PEG2000	2.5
34	6D8i	35	DOPE	16	46.5	C14-PEG2000	2.5
35	6D18	35	DOPE	16	46.5	C14-PEG2000	2.5
36	6D9.2	35	DOPE	16	46.5	C14-PEG2000	2.5
37	7D4	35	DOPE	16	46.5	C14-PEG2000	2.5
38	7D6.2	35	DOPE	16	46.5	C14-PEG2000	2.5
39	7D8	35	DOPE	16	46.5	C14-PEG2000	2.5
40	7D8i	35	DOPE	16	46.5	C14-PEG2000	2.5
41	7D18	35	DOPE	16	46.5	C14-PEG2000	2.5
42	7D9.2	35	DOPE	16	46.5	C14-PEG2000	2.5
43	8D4	35	DOPE	16	46.5	C14-PEG2000	2.5
44	8D6.2	35	DOPE	16	46.5	C14-PEG2000	2.5
45	8D8	35	DOPE	16	46.5	C14-PEG2000	2.5
46	8D8i	35	DOPE	16	46.5	C14-PEG2000	2.5
47	8D18	35	DOPE	16	46.5	C14-PEG2000	2.5
48	8D9.2	35	DOPE	16	46.5	C14-PEG2000	2.5
49	10D4	35	DOPE	16	46.5	C14-PEG2000	2.5
50	10D6.2	35	DOPE	16	46.5	C14-PEG2000	2.5
51	10D8	35	DOPE	16	46.5	C14-PEG2000	2.5
52	10D8i	35	DOPE	16	46.5	C14-PEG2000	2.5
53	10D18	35	DOPE	16	46.5	C14-PEG2000	2.5
54	10D9.2	35	DOPE	16	46.5	C14-PEG2000	2.5
55	11D4	35	DOPE	16	46.5	C14-PEG2000	2.5
56	11D6.2	35	DOPE	16	46.5	C14-PEG2000	2.5
57	11D8	35	DOPE	16	46.5	C14-PEG2000	2.5
58	11D8i	35	DOPE	16	46.5	C14-PEG2000	2.5
59	11D18	35	DOPE	16	46.5	C14-PEG2000	2.5
60	11D9.2	35	DOPE	16	46.5	C14-PEG2000	2.5
61	13D4	35	DOPE	16	46.5	C14-PEG2000	2.5
62	13D6.2	35	DOPE	16	46.5	C14-PEG2000	2.5
63	13D8	35	DOPE	16	46.5	C14-PEG2000	2.5
64	13D8i	35	DOPE	16	46.5	C14-PEG2000	2.5

Index	Ionizable lipid	%mol	Helper lipid	%mol	Cholesterol (%mol)	PEG-lipid	%mol
65	13D18	35	DOPE	16	46.5	C14-PEG2000	2.5
66	13D9.2	35	DOPE	16	46.5	C14-PEG2000	2.5
67	12D4	35	DOPE	16	46.5	C14-PEG2000	2.5
68	12D6.2	35	DOPE	16	46.5	C14-PEG2000	2.5
69	12D8	35	DOPE	16	46.5	C14-PEG2000	2.5
70	12D8i	35	DOPE	16	46.5	C14-PEG2000	2.5
71	12D18	35	DOPE	16	46.5	C14-PEG2000	2.5
72	12D9.2	35	DOPE	16	46.5	C14-PEG2000	2.5
73	14D4	35	DOPE	16	46.5	C14-PEG2000	2.5
74	14D6.2	35	DOPE	16	46.5	C14-PEG2000	2.5
75	14D8	35	DOPE	16	46.5	C14-PEG2000	2.5
76	14D8i	35	DOPE	16	46.5	C14-PEG2000	2.5
77	14D18	35	DOPE	16	46.5	C14-PEG2000	2.5
78	14D9.2	35	DOPE	16	46.5	C14-PEG2000	2.5
79	15D4	35	DOPE	16	46.5	C14-PEG2000	2.5
80	15D6.2	35	DOPE	16	46.5	C14-PEG2000	2.5
81	15D8	35	DOPE	16	46.5	C14-PEG2000	2.5
82	15D8i	35	DOPE	16	46.5	C14-PEG2000	2.5
83	15D18	35	DOPE	16	46.5	C14-PEG2000	2.5
84	15D9.2	35	DOPE	16	46.5	C14-PEG2000	2.5
85	16D4	35	DOPE	16	46.5	C14-PEG2000	2.5
86	16D6.2	35	DOPE	16	46.5	C14-PEG2000	2.5
87	16D8	35	DOPE	16	46.5	C14-PEG2000	2.5
88	16D8i	35	DOPE	16	46.5	C14-PEG2000	2.5
89	16D18	35	DOPE	16	46.5	C14-PEG2000	2.5
90	16D9.2	35	DOPE	16	46.5	C14-PEG2000	2.5
91	17D4	35	DOPE	16	46.5	C14-PEG2000	2.5
92	17D6.2	35	DOPE	16	46.5	C14-PEG2000	2.5
93	17D8	35	DOPE	16	46.5	C14-PEG2000	2.5
94	17D8i	35	DOPE	16	46.5	C14-PEG2000	2.5
95	17D18	35	DOPE	16	46.5	C14-PEG2000	2.5
96	17D9.2	35	DOPE	16	46.5	C14-PEG2000	2.5
97	18D4	35	DOPE	16	46.5	C14-PEG2000	2.5
98	18D6.2	35	DOPE	16	46.5	C14-PEG2000	2.5
99	18D8	35	DOPE	16	46.5	C14-PEG2000	2.5
100	18D8i	35	DOPE	16	46.5	C14-PEG2000	2.5
101	18D18	35	DOPE	16	46.5	C14-PEG2000	2.5
102	18D9.2	35	DOPE	16	46.5	C14-PEG2000	2.5
103	19D4	35	DOPE	16	46.5	C14-PEG2000	2.5
104	19D6.2	35	DOPE	16	46.5	C14-PEG2000	2.5
105	19D8	35	DOPE	16	46.5	C14-PEG2000	2.5
106	19D8i	35	DOPE	16	46.5	C14-PEG2000	2.5
107	19D18	35	DOPE	16	46.5	C14-PEG2000	2.5
108	19D9.2	35	DOPE	16	46.5	C14-PEG2000	2.5
109	20D4	35	DOPE	16	46.5	C14-PEG2000	2.5
110	20D6.2	35	DOPE	16	46.5	C14-PEG2000	2.5
111	20D8	35	DOPE	16	46.5	C14-PEG2000	2.5
112	20D8i	35	DOPE	16	46.5	C14-PEG2000	2.5
113	20D18	35	DOPE	16	46.5	C14-PEG2000	2.5
114	20D9.2	35	DOPE	16	46.5	C14-PEG2000	2.5
115	9D4	35	DOPE	16	46.5	C14-PEG2000	2.5
116	9D6.2	35	DOPE	16	46.5	C14-PEG2000	2.5
117	9D8	35	DOPE	16	46.5	C14-PEG2000	2.5
118	9D8i	35	DOPE	16	46.5	C14-PEG2000	2.5
119	9D18	35	DOPE	16	46.5	C14-PEG2000	2.5
120	9D9.2	35	DOPE	16	46.5	C14-PEG2000	2.5
121	C12-200	35	DOPE	16	46.5	C14-PEG2000	2.5
122	cKK-E12	35	DOPE	16	46.5	C14-PEG2000	2.5
123	DLin-MC3-DMA	50	DSPC	10	38.5	DMG-PEG2000	1.5
124	306Oi10	35	DOPE	16	46.5	C14-PEG2000	2.5
125	SM-102	50	DSPC	10	38.5	DMG-PEG2000	1.5
126	ALC-0315	46.3	DSPC	9.4	42.7	ALC-0159	1.6
127	C14-482	35	DOPE	16	46.5	C14-PEG2000	2.5
128	C14-488	35	DOPE	16	46.5	C14-PEG2000	2.5

Index	Ionizable lipid	%mol	Helper lipid	%mol	Cholesterol (%mol)	PEG-lipid	%mol
129	C16-488	35	DOPE	16	46.5	C14-PEG2000	2.5
130	C12-494	35	DOPE	16	46.5	C14-PEG2000	2.5
131	C14-494	35	DOPE	16	46.5	C14-PEG2000	2.5
132	C16-494	35	DOPE	16	46.5	C14-PEG2000	2.5
133	C14-c494	35	DOPE	16	46.5	C14-PEG2000	2.5

Supplementary Table 3: Formulation details of tested LNP library.

Marker	Clone
CD3	17A2
CD19	6D5
CD11b	M1/70
CD11c	N418
CD31	MEC13.3
CD45	30-F11
CD68	FA-11
CD146	ME-9F1
CD326	G8.8
CD335	29A1.4
F4/80	BM8

Supplementary Table 4: Antibody clones used for flow cytometry and FACS.

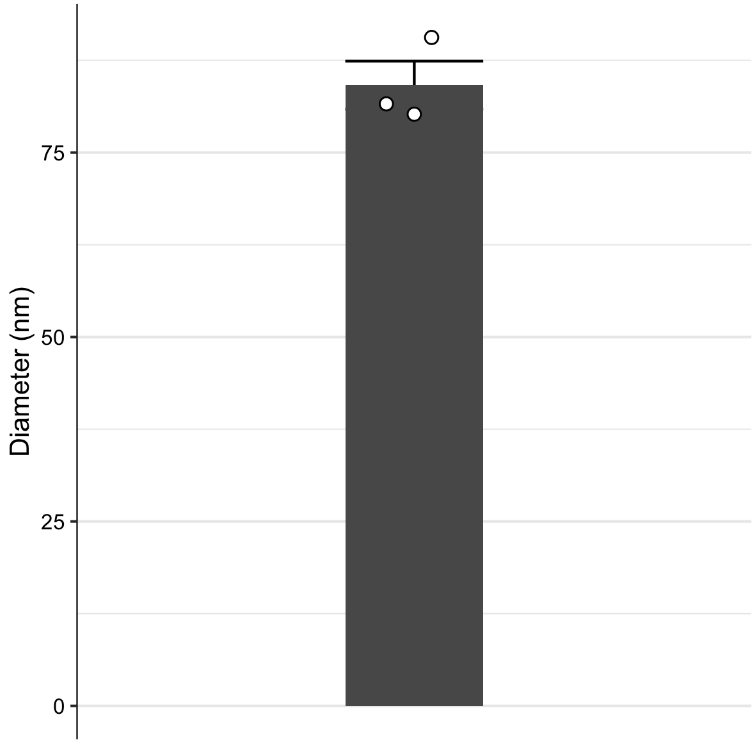

Supplementary Figure 1: Hydrodynamic diameter of C12-494 LNPs containing b-mRNA pool as measured by DLS.

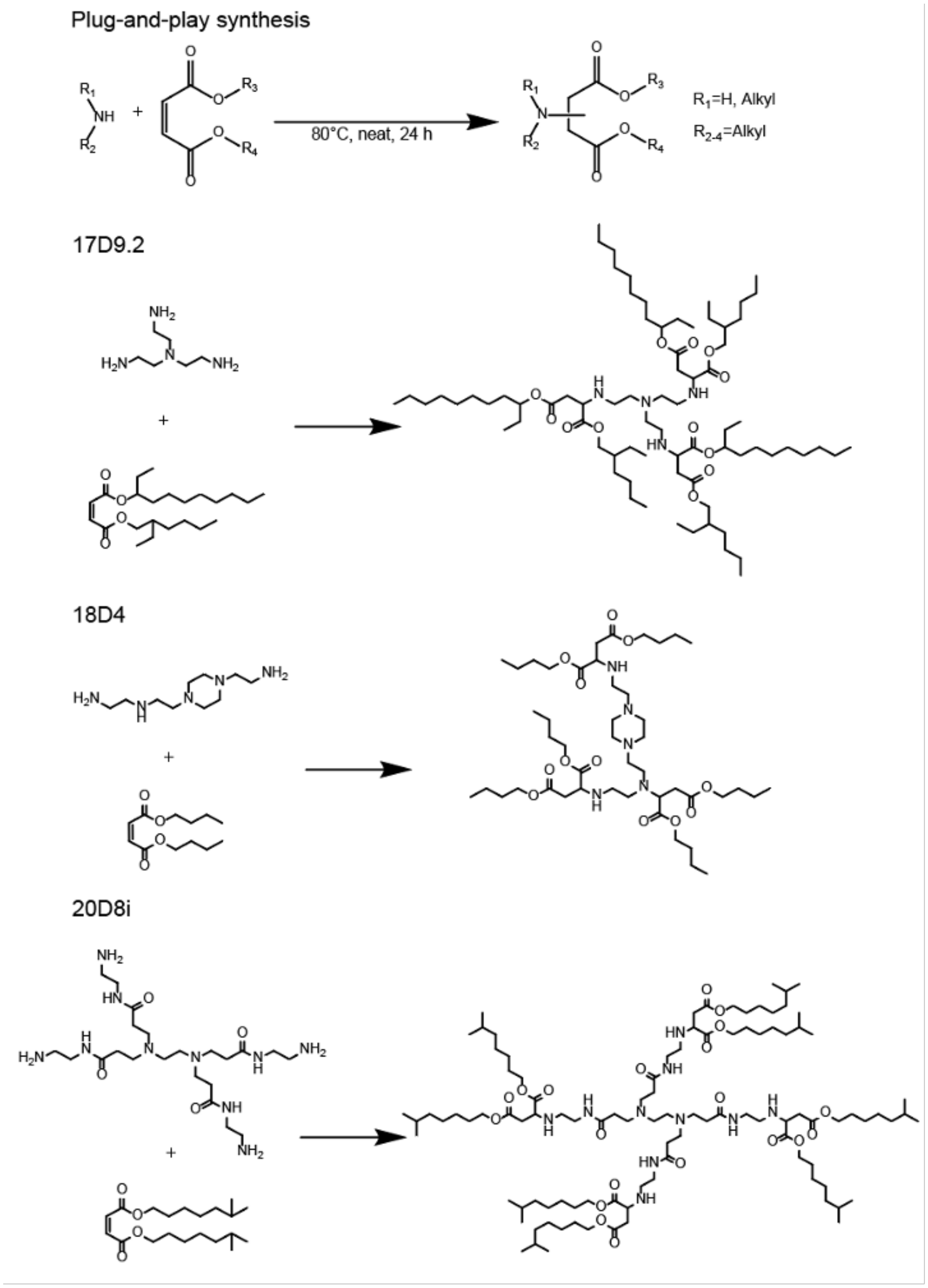

Supplementary Figure 2: Plug-and-play reaction principle. Schematic of the one-step Michael addition between amines/thiols and dialkyl maleates used to assemble ionizable lipids under neat conditions (80 °C, 24 h). Dialkyl maleates provide higher reactivity and favor mono-addition, facilitating plug-and-play diversification; three example routes (17D9.2, 18D4, and 20D8i) are shown.

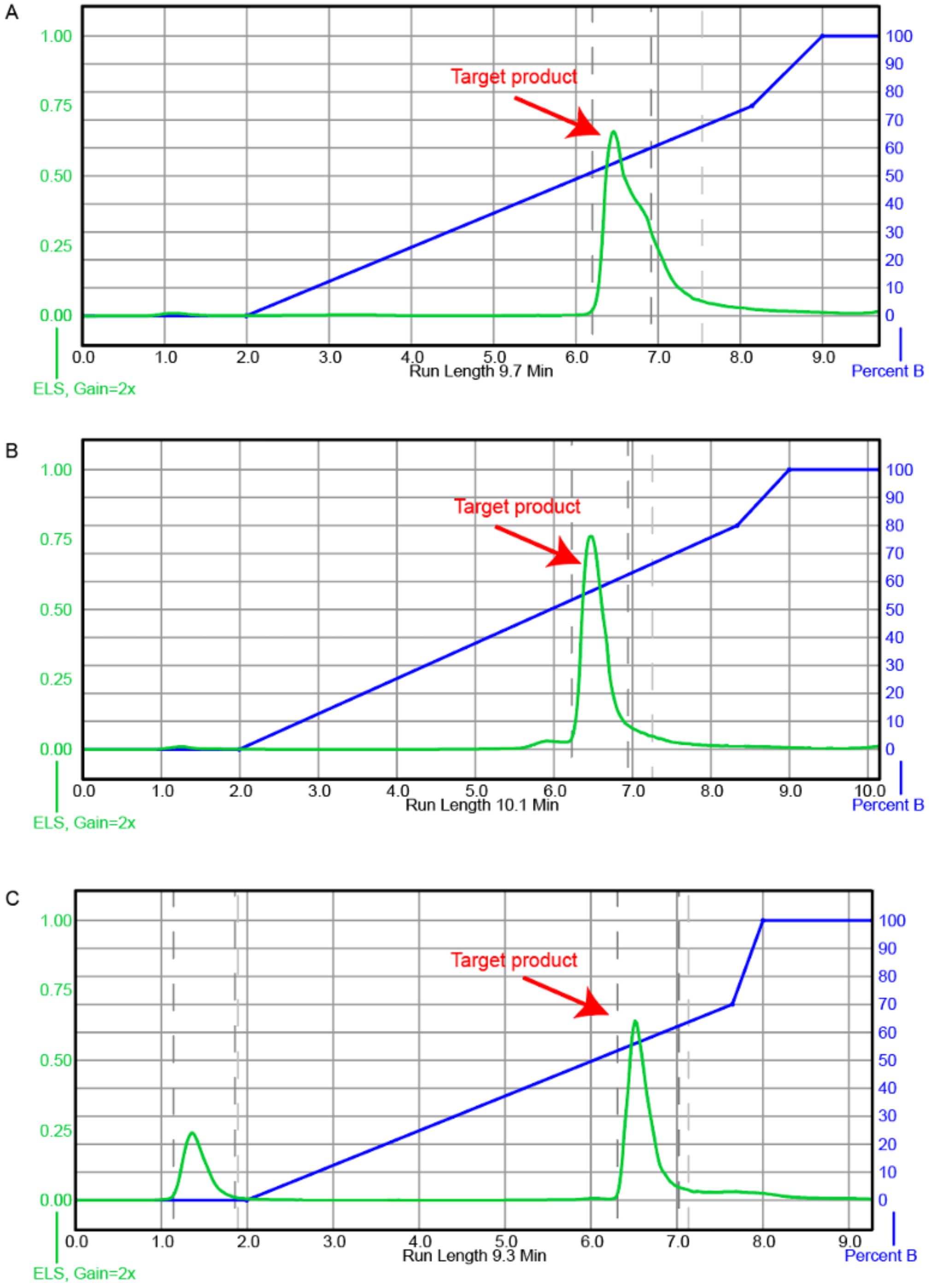

Supplementary Figure 3: Representative flash purification traces (ELS detection). CombiFlash NextGen 300+ traces for 17D9.2 (A), 18D4 (B), and 20D8i (C) ionizable lipids. Crude products were purified with a 10-min gradient from 100% CH_2Cl_2 to CH_2Cl_2/MeOH/NH_4OH (75:22:3), monitored by an evaporative light-scattering detector (ELS). Target products elute as single symmetric peaks.

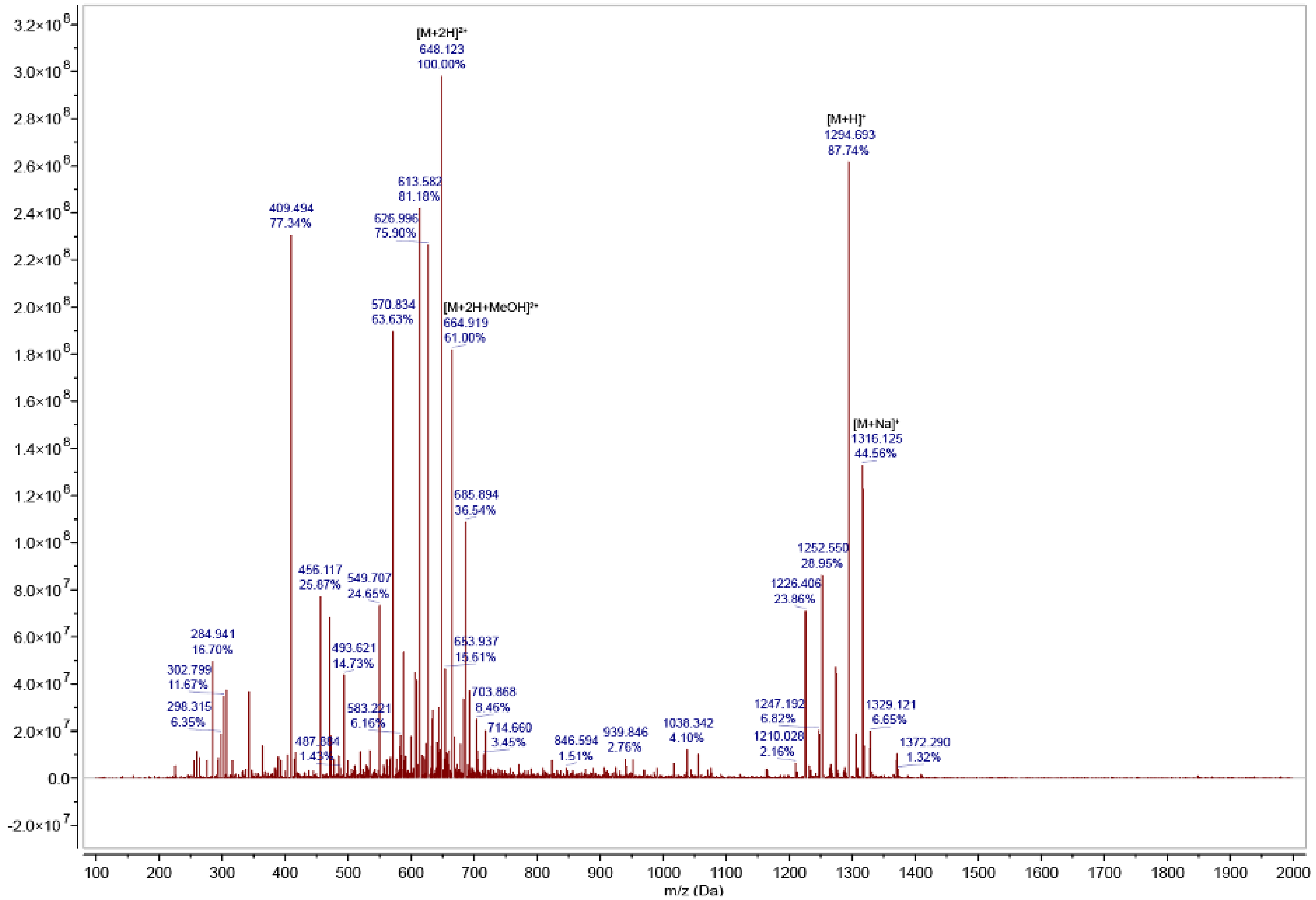

Supplementary Figure 4: Mass spectrum of 17D9.2 (ESI, loop injection).

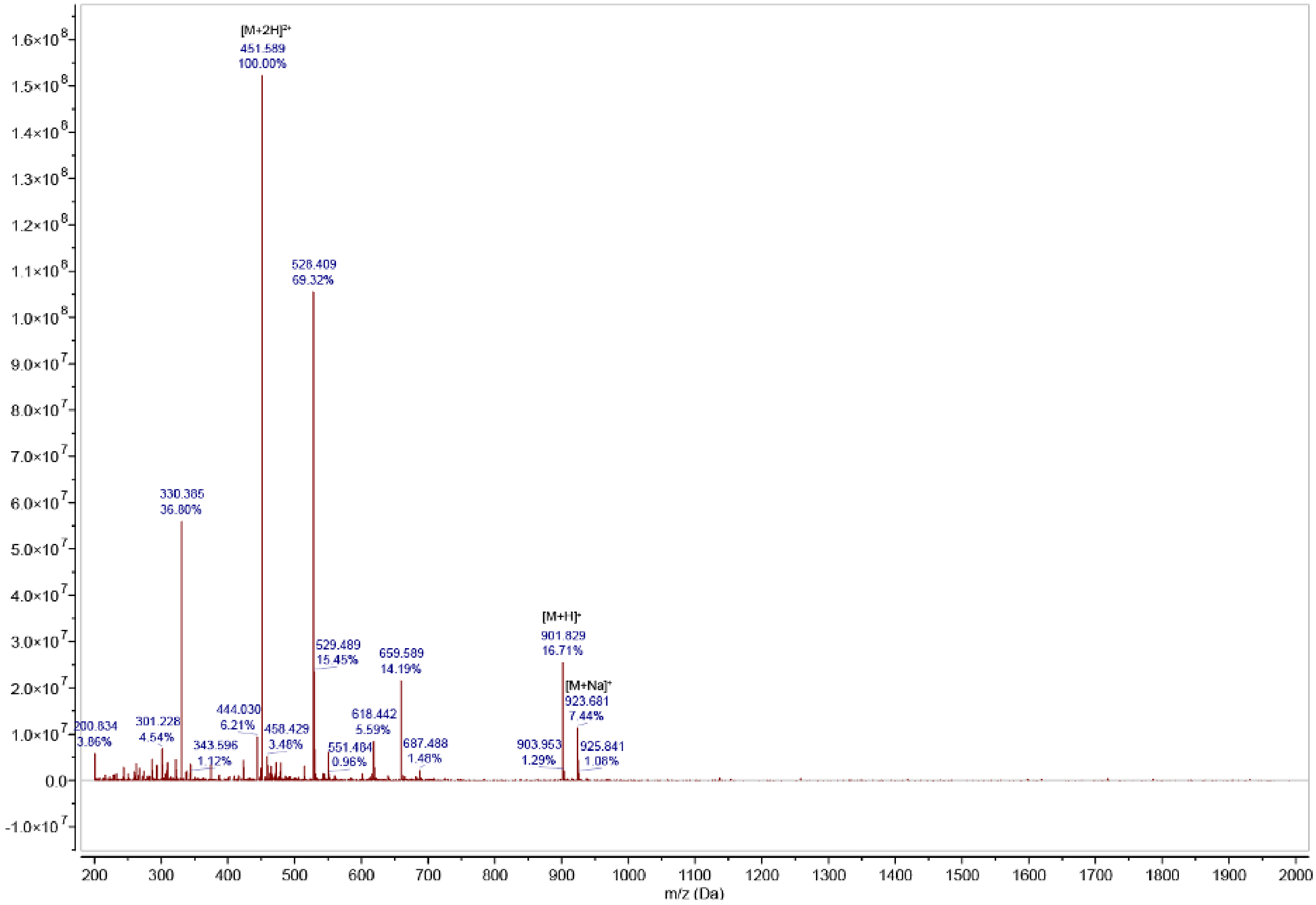

Supplementary Figure 5: Mass spectrum of 18D4 (ESI, loop injection).

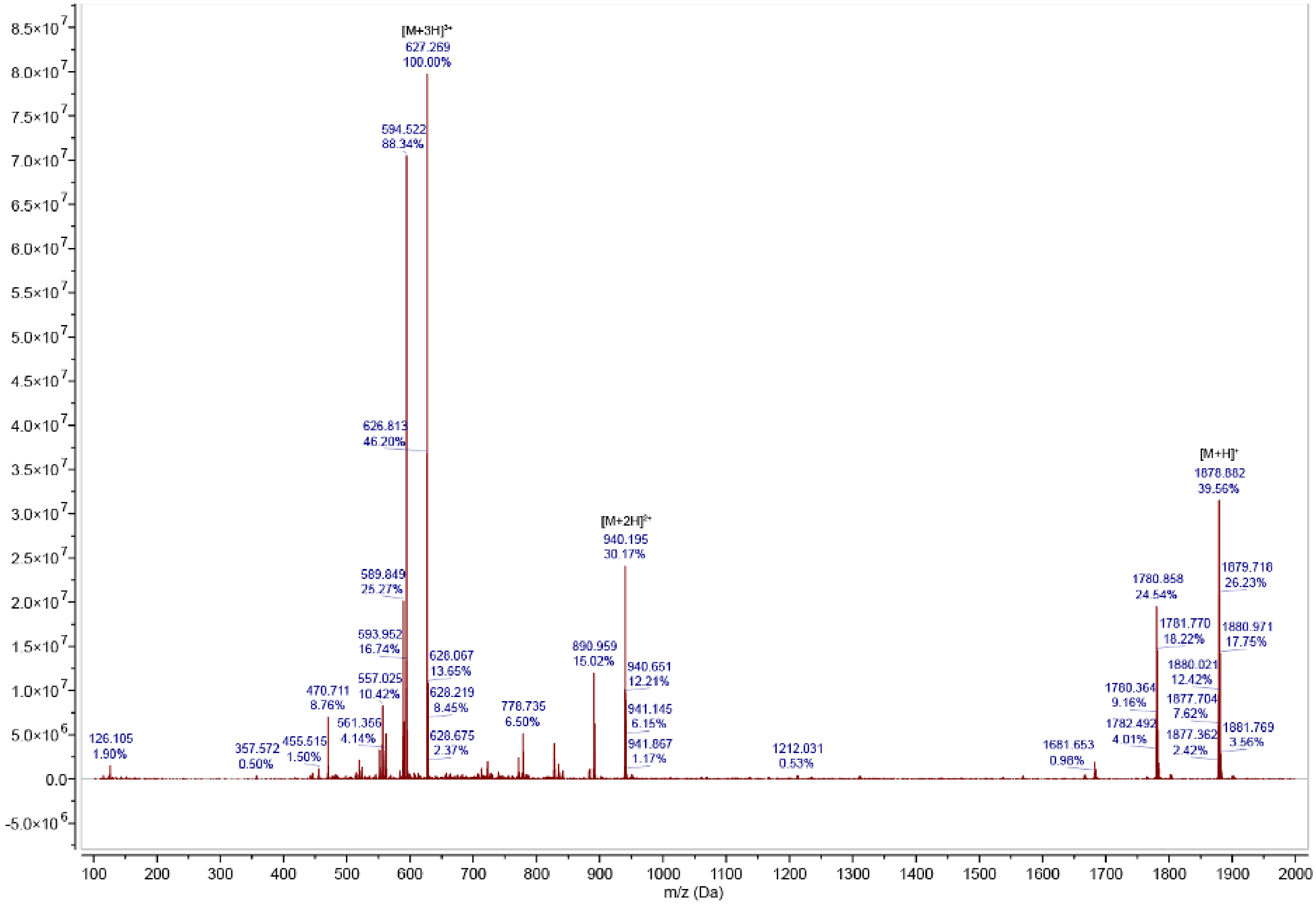

Supplementary Figure 6: Mass spectrum of 20D8i (ESI, loop injection).

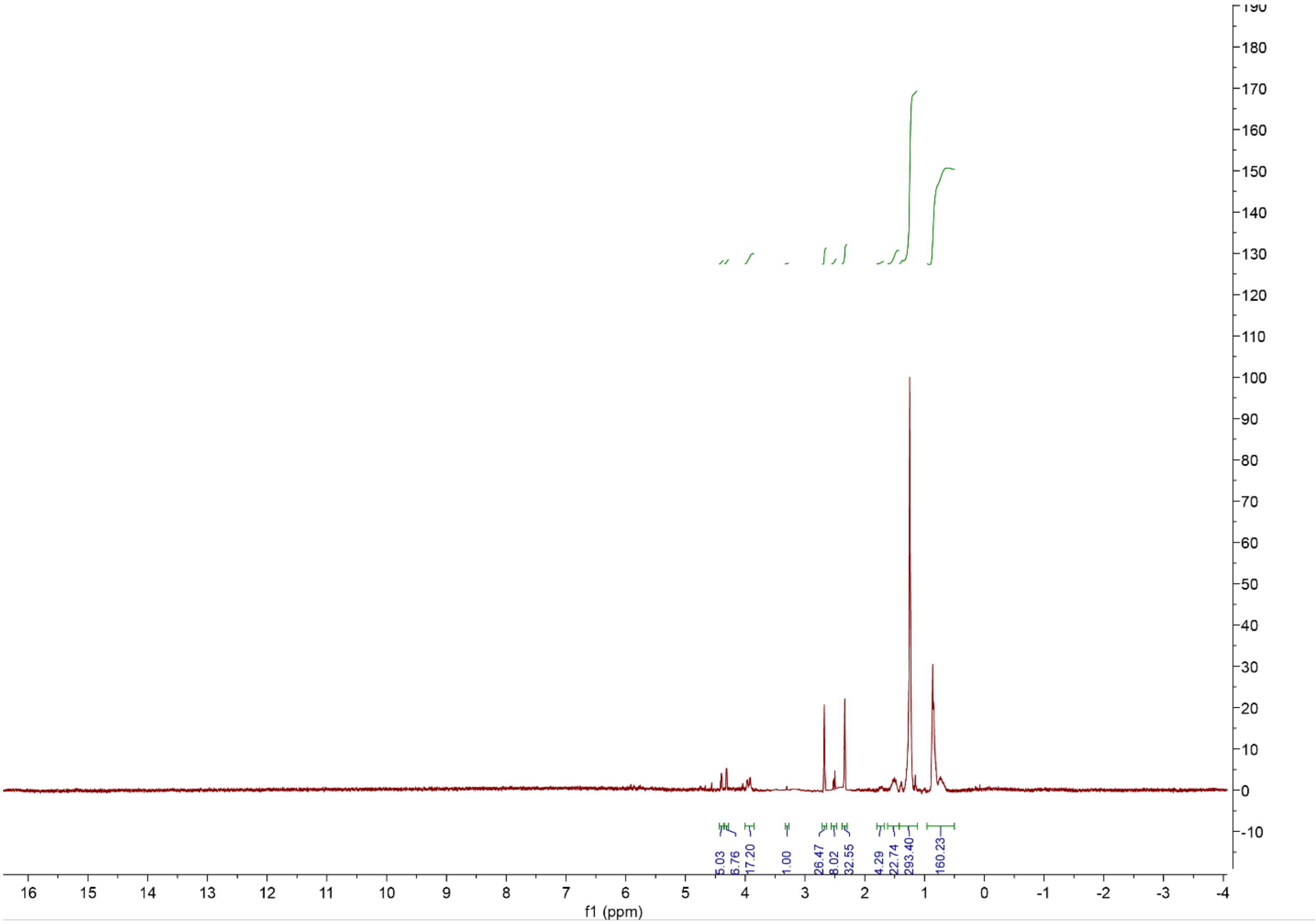

Supplementary Figure 7: ^{1}H NMR (400 MHz) spectrum of 17D9.2 in $CDCl_3$.

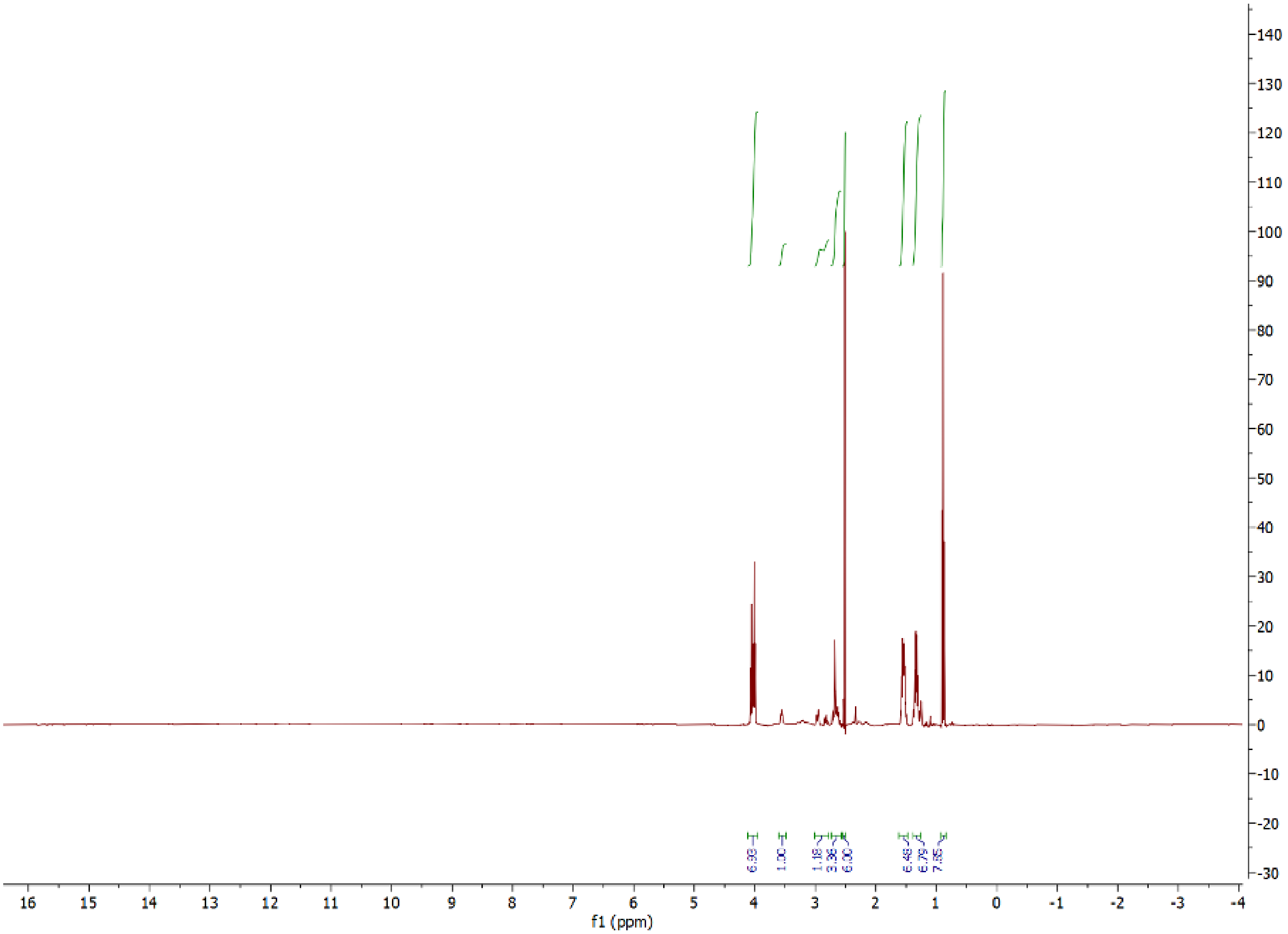

Supplementary Figure 8: ^{1}H NMR (400 MHz) spectrum of 18D4 in $CDCl_3$.

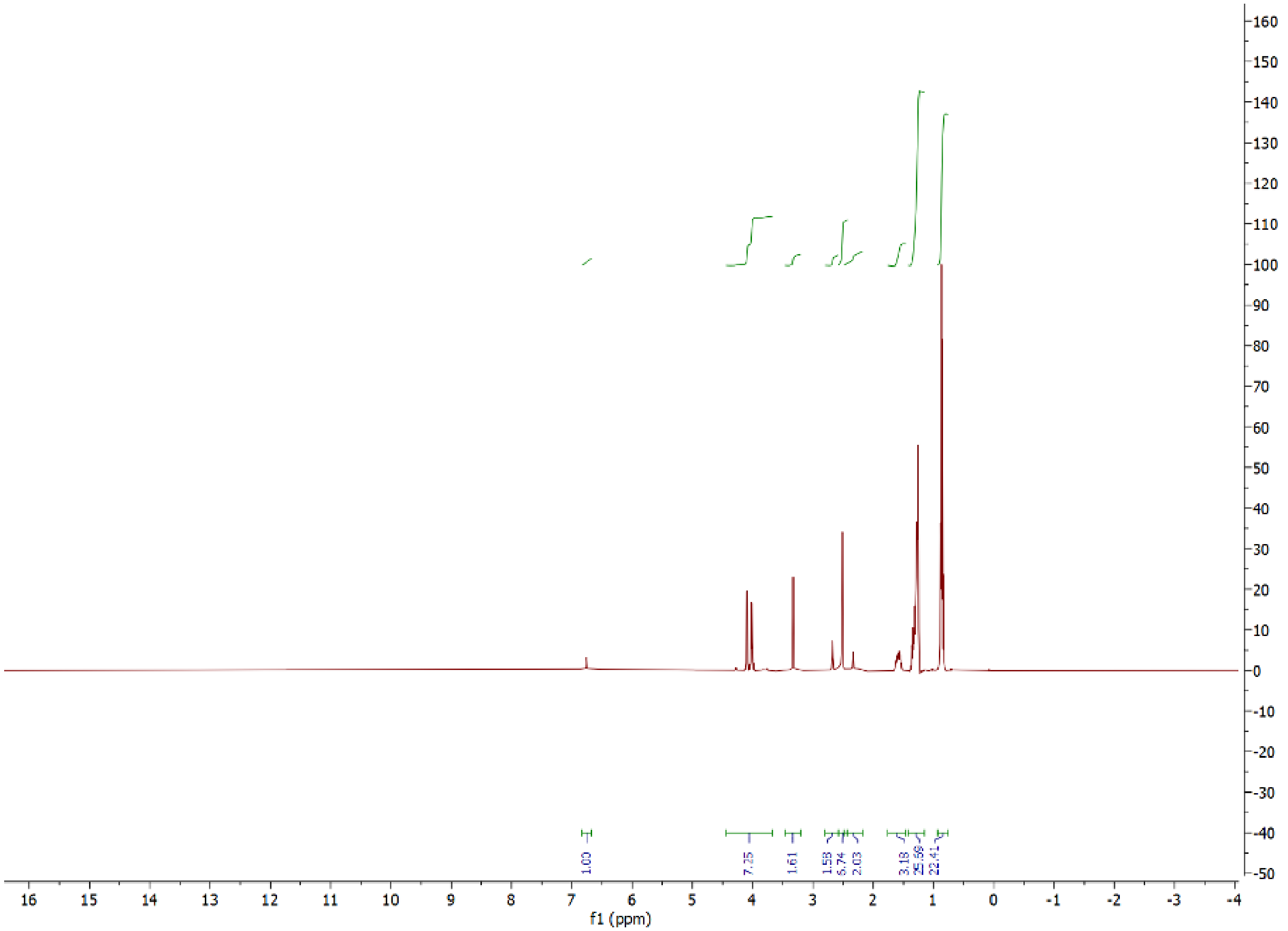

Supplementary Figure 9: ^{1}H NMR (400 MHz) spectrum of 20D8i in $CDCl_3$.

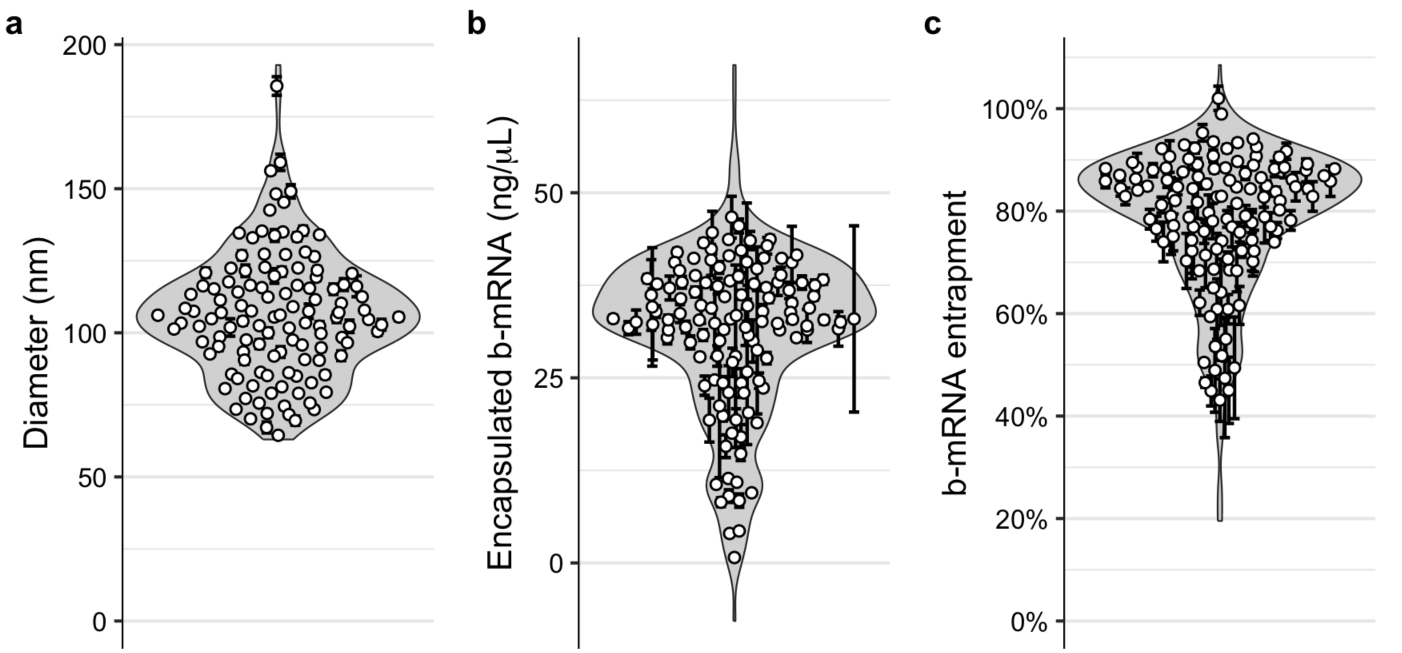

Supplementary Figure 10: Physicochemical characterization of b-mRNA LNP library. **a.** Hydrodynamic LNP diameter as measured by DLS. **b.** Encapsulated b-mRNA concentration as measured by RiboGreen. **c.** b-mRNA entrapment efficiency as measured by RiboGreen. Data are presented as mean ± standard error of the mean from $n \geq 4$ independent measurements.

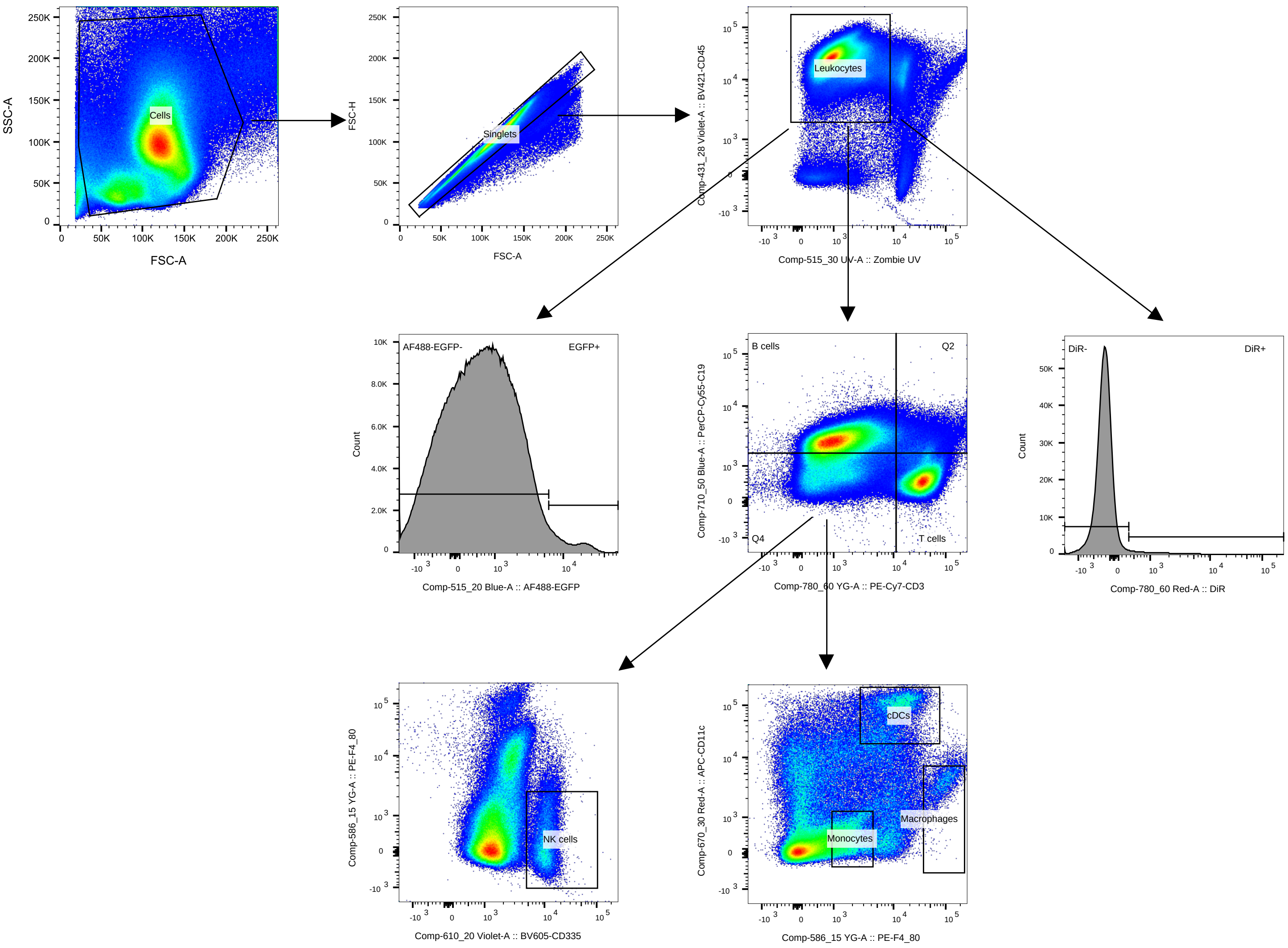

Supplementary Figure 11: Representative blood/spleen flow cytometry gating scheme used for initial FACS-based b-mRNA LNP screen and for subsequent validation experiments.

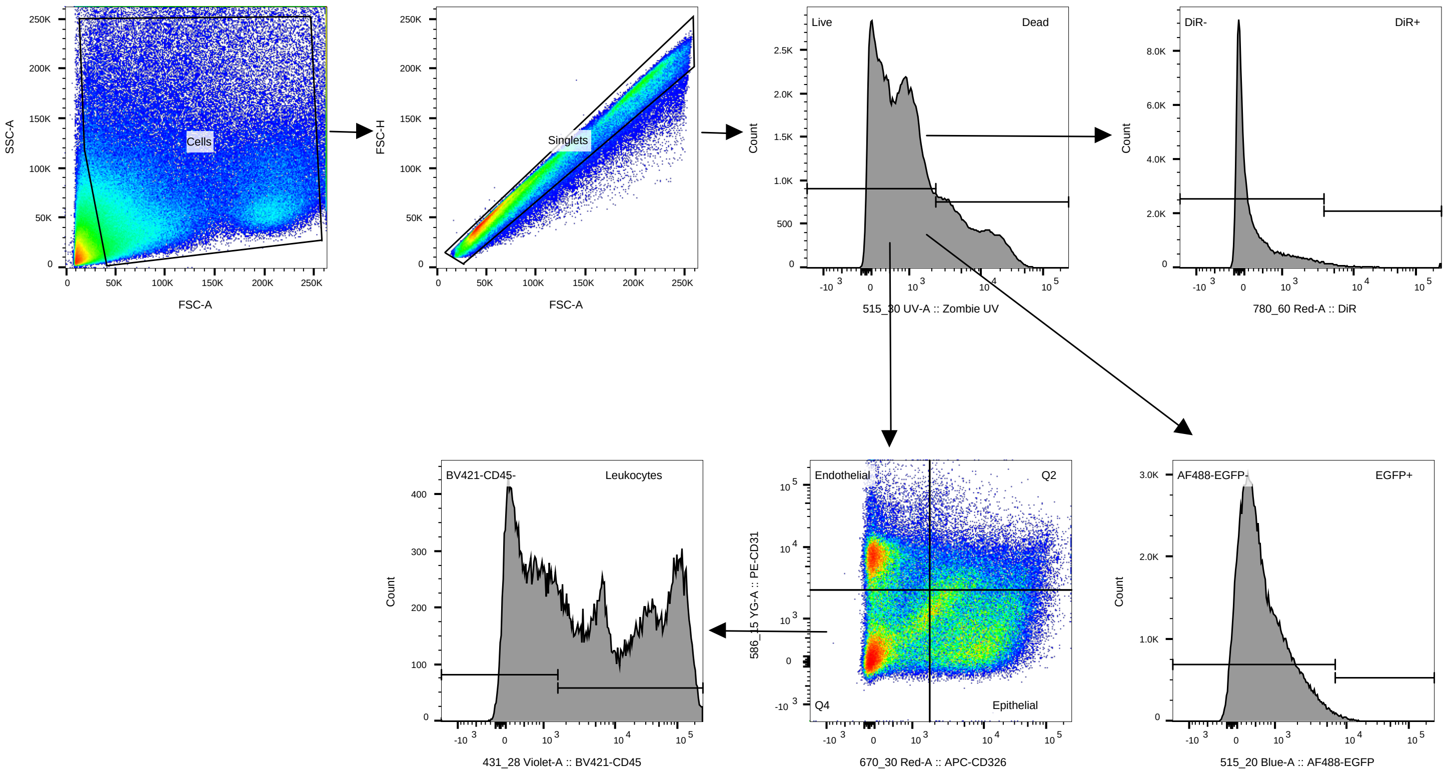

Supplementary Figure 12: Representative lung flow cytometry gating scheme used for initial FACS-based b-mRNA LNP screen and for subsequent validation experiments.

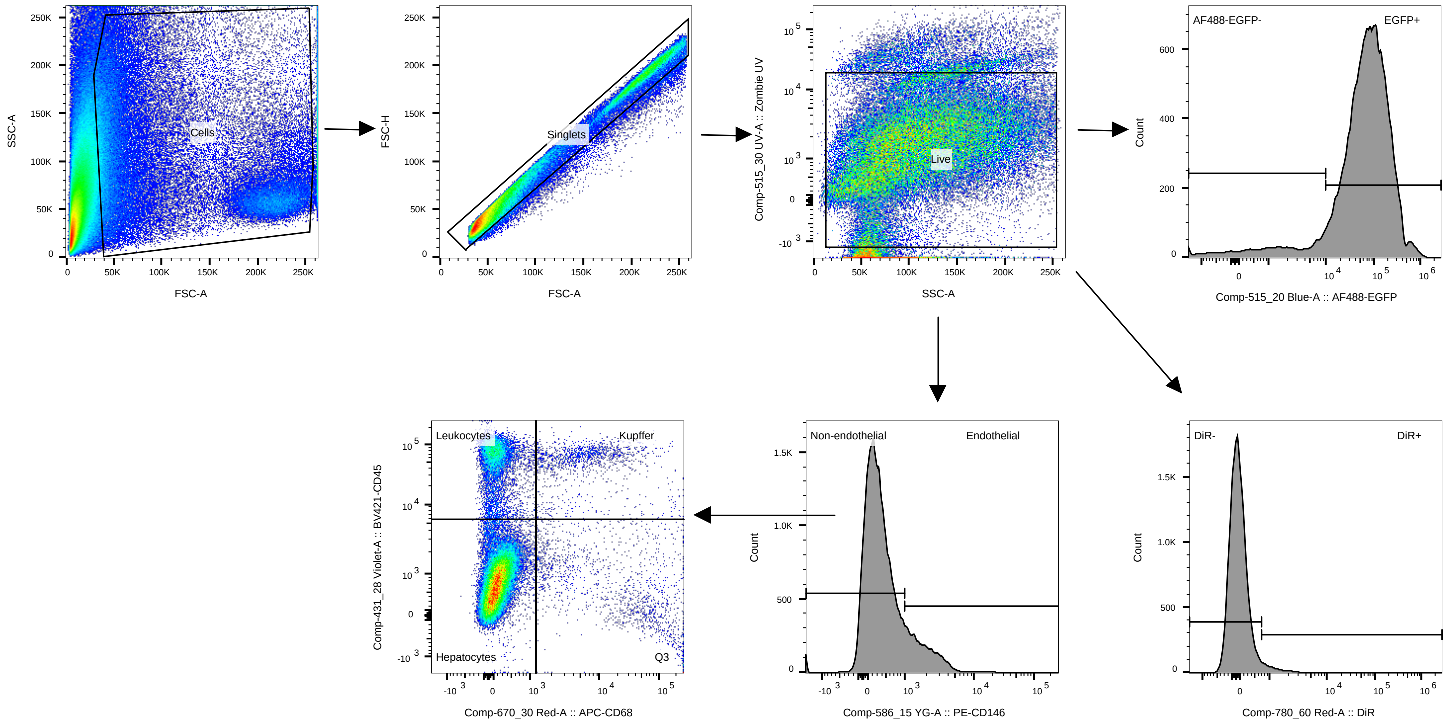

Supplementary Figure 13: Representative liver flow cytometry gating scheme used for initial FACS-based b-mRNA LNP screen and for subsequent validation experiments.

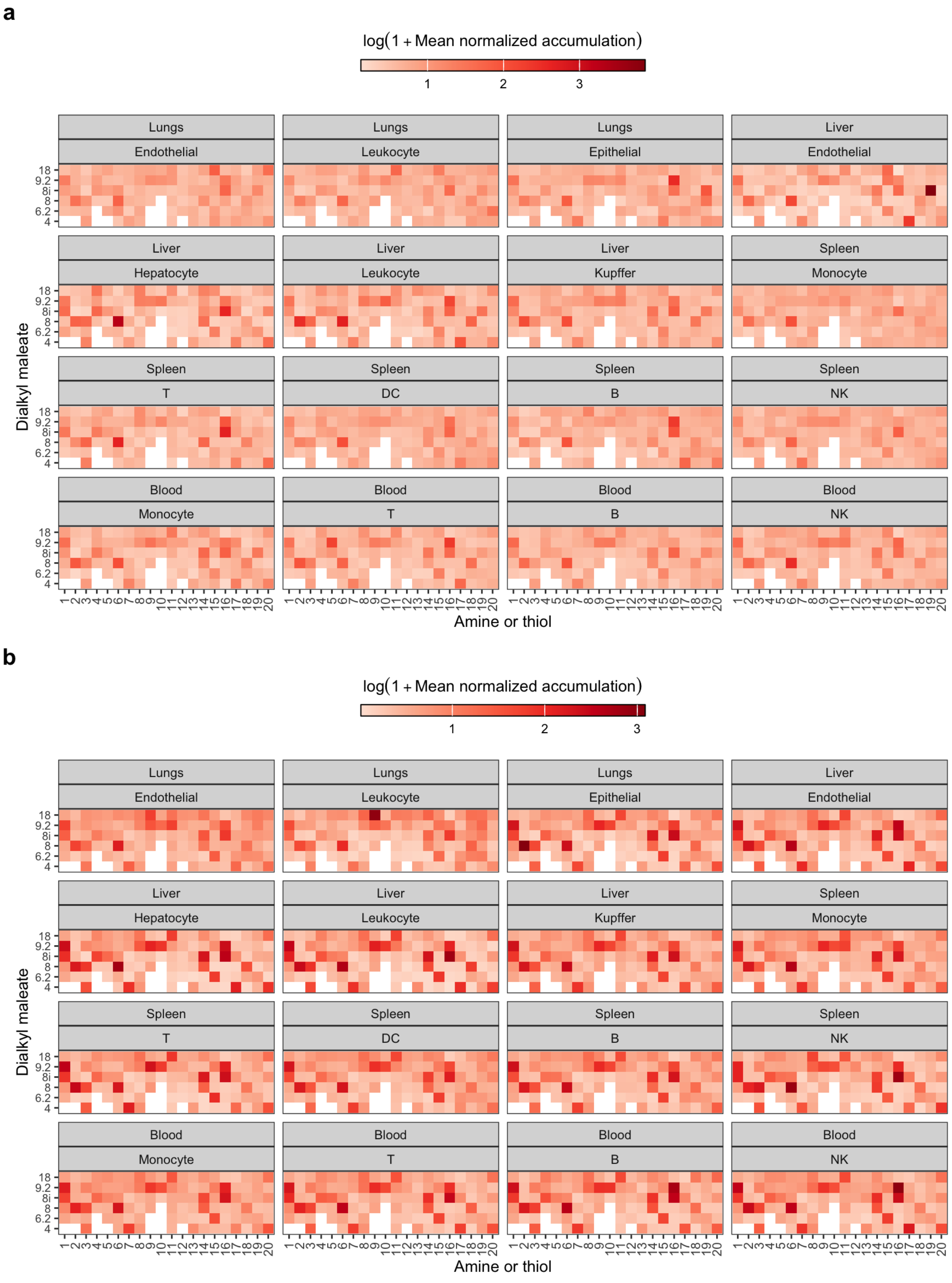

Supplementary Figure 14: Ionizable lipid structure-function relationship identified by HTS for C57BL/6 (**a**) and *APOE*$^{-/-}$ (**b**) mouse strains. Normalized accumulation is indicated for each organ and cell type combination analyzed.

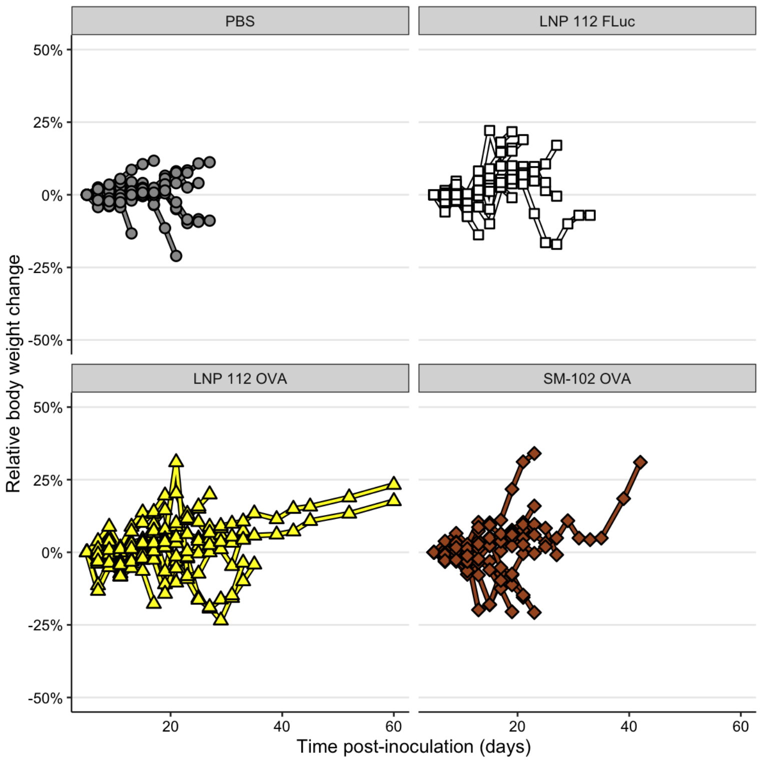

Supplementary Figure 15: Body weight change of mice inoculated with B16-OVA melanoma cells and given the indicated treatments in a therapeutic cancer vaccine model.

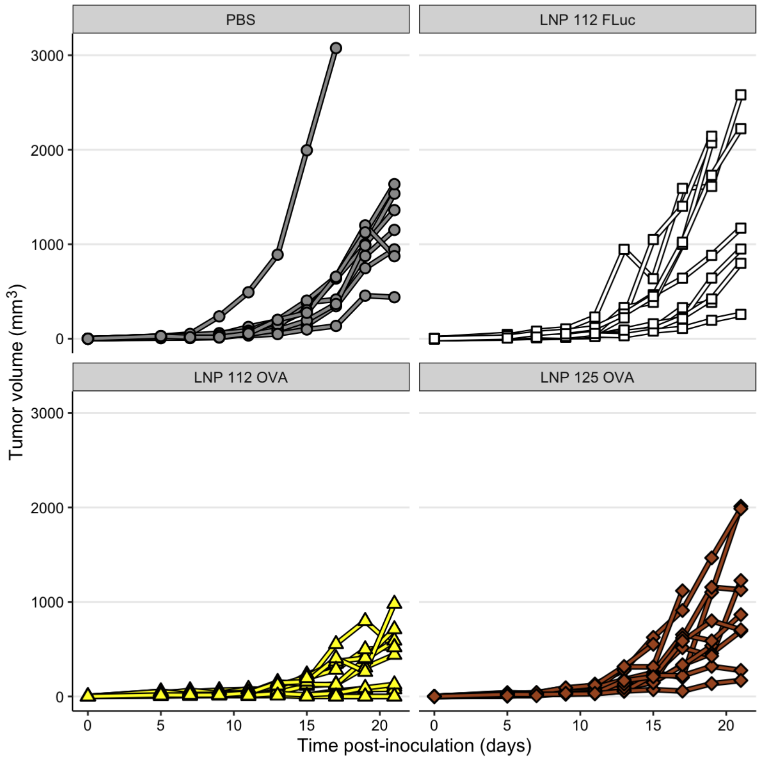

Supplementary Figure 16: Tumor growth curves from mice inoculated with B16-OVA melanoma cells and given the indicated treatments in a therapeutic cancer vaccine model.

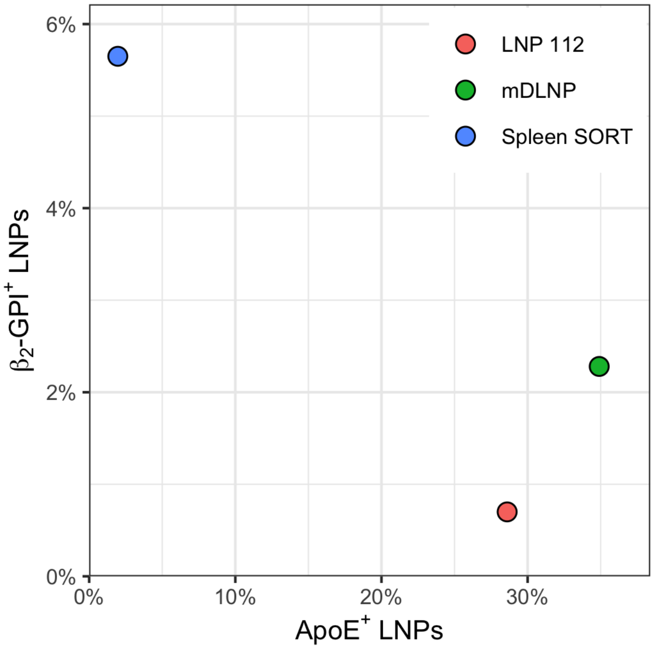

Supplementary Figure 17: Summary of protein adsorption to selective organ targeting (SORT) lipid nanoparticles (LNPs) as measured by small particle flow cytometry. Consistent with previous reports (DOI: 10.1073/pnas.2109256118), the addition of anionic phospholipid to mDLNP to produce spleen SORT LNPs results in a marked decrease in apolipoprotein E (ApoE) adsorption and an increase in β_2-glycoprotein 1 (β_2-GPI) adsorption.
